# The genus *Rachicladosporium*: introducing new species from sooty mould communities and excluding cold adapted species

**DOI:** 10.1038/s41598-023-49696-9

**Published:** 2023-12-21

**Authors:** Marcin Piątek, Monika Stryjak-Bogacka, Paweł Czachura, Magdalena Owczarek-Kościelniak

**Affiliations:** 1 W. Szafer Institute of Botany, Polish Academy of Sciences, Lubicz 46, 31-512 Kraków, Poland; 2 Present Address: Norwegian Veterinary Institute, P.O. Box 64, 1431 Ås, Norway

**Keywords:** Evolution, Microbiology

## Abstract

The fungal genus *Rachicladosporium* (*Cladosporiales*, *Cladosporiaceae*), typified by cladosporium-like *Rachicladosporium luculiae*, includes a morphologically diverse assemblage of species. The species of this genus were reported from different substrates, habitats and environments, including plant leaves and needles, twig, black mould on baobab trees, rocks and insects. In this study, four new *Rachicladosporium* species (*R. europaeum*, *R. ignacyi*, *R. kajetanii*, *R. silesianum*) isolated from sooty mould communities covering leaves and needles of trees and shrubs in Poland are described. The new species are delineated based on morphological characteristics and molecular phylogenetic analyses using concatenated ITS, LSU, and *rpb2* sequences. All newly described species are nested in the main *Rachicladosporium* lineage (centred around the type species), which contains species that are able to grow at 25 °C. By contrast, four cold adapted, endolithic species known from Antarctica (*R. antarcticum*, *R. aridum*, *R. mcmurdoi*) and Italian Alps (*R. monterosanum*) form distant phylogenetic lineage and do not grow at this temperature. Therefore, they are accommodated in the new genus *Cryoendolithus*, typified by *Cryoendolithus mcmurdoi*.

## Introduction

The genus *Rachicladosporium* resides in the family *Cladosporiaceae* (= *Davidiellaceae*) and broadly treated order *Capnodiales*^1–5^ or narrowly treated order *Cladosporiales*^6^. The genus was introduced to accommodate the single cladosporium-like species *Rachicladosporium luculiae* isolated from leaf spots on *Luculia* sp. (*Rubiaceae*) in New Zealand and characterised by having “an apical conidiophore rachis with inconspicuous to subconspicuous scars and unthickened, not darkened-refractive conidial hila”^7^. The apical conidiophore rachis was not observed in four other cladosporium-like species: *Rachicladosporium americanum* and *Rachicladosporium cboliae* described from leaf litter and twig debris in the USA, respectively, *Rachicladosporium pini* described from needles of *Pinus monophylla* in the Netherlands, and *Rachicladosporium africanum* described from black mould on the bark of baobab *Adansonia digitata* in South Africa^2,8–10^, and is therefore considered not diagnostic at the genus level^8^. In contrast to those five species having asexual cladosporium-like morph, two other *Rachicladosporium* species produce a sexual mycosphaerella-like morph. These are: *Rachicladosporium eucalypti* described from leaves of *Eucalyptus globulus* in Ethiopia^11^ and *Rachicladosporium iridis* described from leaves of *Iris pumila* in Germany and epitypified from material on leaf of *Iris pseudacorus* in the Netherlands^12^. The other species, *Rachicladosporium corymbiae* described from leaf spots of *Corymbia citriodora* in Ghana, produces both the asexual cladosporium-like and sexual mycosphaerella-like morphs^13^. Yet another group of *Rachicladosporium* species is known to produce only sterile mycelium, with or without chlamydospores/arthroconidia. This group includes seven species isolated from rocks in the coldest, extreme environments of the world: *Rachicladosporium antarcticum*, *R. aridum* and *R. mcmurdoi* described from Antarctica^14–16^ and *R. alpinum*, *R. inconspicuum*, *R. monterosanum*, and *R. paucitum* described from the Italian Alps^14,15^. Summarizing, currently 14 species producing an asexual cladosporium-like morph, mycosphaerella-like sexual morph or only sterile mycelium with or without chlamydospores/arthroconidia are accommodated in the genus *Rachicladosporium*.

The recognized species (i.e. named) of the genus *Rachicladosporium* were reported from different substrates, habitats and environments, including plant leaves and needles, twig, black mould on baobab trees, rocks and *Scolytus multistriatus* beetles^2,7–20^. Unclassified *Rachicladosporium* species were also reported from other substrates, for example as endophytes of different *Pinus* species and *Angelica sinensis*^21–23^, from *Scolytus multistriatus* and *Tomicus brevipilosus* beetles^17,24^, bark of avocado trees^25^ or rhizosphere soil and plant tissues of *Ligustrum lucidum*^26^.

Phylogenetic relationships within the genus *Rachicladosporium* were analysed at several occasions, however with conflicted results, especially regarding the position of rock inhabiting species from Antarctica and Italian Alps (i.e. group of *R. antarcticum*, *R. aridum*, *R. mcmurdoi* and *R. monterosanum*). They were either paraphyletic^10,14,16^, sister to^19^ or intermixed with remaining *Rachicladosporium* species^14^ in different phylogenetic analyses. Draft genome sequences were reported for two Antarctic endolithic species: *Rachicladosporium antarcticum* CCFEE 5527 and *Rachicladosporium* sp. CCFEE 5018 (probably still undescribed species)^27^. The genome analysis of *Rachicladosporium antarcticum* showed a high portfolio of enzymes, which may be related to occurrence of this fungus in organic-poor habitats and necessity to degrade everything that is available^28^.

Sooty moulds are special fungi that live on honeydew of aphids or sweet plant exudates. They usually form multispecies communities on the surface of leaves or needles of angiosperm and gymnosperm woody plants, without host penetration. The species composition of these communities is still weakly known, especially in temperate climate regions. The available data suggest that taxonomic assignment of temperate climate sooty moulds is different from tropical and subtropical sooty moulds^6,29–33^. The species of the genus *Rachicladosporium* were previously not reported as inhabitants of sooty mould communities.

Here, four new *Rachicladosporium* species isolated from sooty mould patches covering leaves and needles of trees and shrubs in Poland are described. All species were delimited based on morphological and molecular phylogenetic analyses. Additionally, four rock inhabiting fungi described from Antarctica and Italian Alps are excluded from the genus *Rachicladosporium* and reclassified in a new genus, based on morphology, temperature preferences and molecular data.

## Materials and methods

### Isolates

Leaves or needles of planted *Acer pseudoplatanus*, *Pinus nigra*, *Quercus robur*, *Taxus baccata*, *Tilia cordata* and *Symphoricarpos albus* covered with sooty moulds were collected in municipal greenery in five cities (Katowice, Kraków, Nowy Sącz, Rzeszów, Tarnów) in southern Poland, secured in a plastic bag and transported to the laboratory. The plant species used in this study are not protected and therefore specific permits for sampling were not required. Within 24 h from the collection, the initial cultures were established. After initial washing of the samples in distilled water, sooty mould colonies were gently scrubbed from the leaves or needles and streaked on three different media: malt extract agar (MEA – Blakeslee's formula), potato dextrose agar (PDA), and rose bengal agar (RBC) (Table 1). The initial cultures were incubated at 15 °C for approximately 1 month and subsequently colonies of growing morphotypes were transferred to the new MEA medium. Subsequently, selected representatives of each morphotype (including representatives from each plant species) were first subjected to molecular analyses employing rDNA ITS sequencing. Next, the morphotypes representing *Rachicladosporium* species were subjected to detailed morphological and molecular analyses using also other DNA markers. Two *Rachicladosporium* strains randomly obtained from leaves of *Betula pendula* and *Rosa* sp. covered with sooty moulds in Nowy Targ County were also included in detailed analyses. Dried specimens obtained from cultures are stored in the fungal collection of the W. Szafer Institute of Botany, Polish Academy of Sciences, Kraków (KRAM F). Cultures are deposited in the culture collection of the Westerdijk Fungal Biodiversity Institute (CBS) and in the W. Szafer Institute of Botany, Polish Academy of Sciences, Kraków. All the methods were carried out in accordance with relevant Institutional guidelines and regulations.

**Table 1:** Microbiological media used for isolations and morphological analyses.

Malt extract agar (MEA)		
Malt extract	20 g/l	Carl Roth, Germany
Glucose	20 g/l	Chempur, Poland
Peptone ex meat	1 g/l	Carl Roth, Germany
Agar	20 g/l	Carl Roth, Germany

Potato dextrose agar (PDA)		
Ready-to-use PDA medium	39 g/l	Carl Roth, Germany

Rose bengal agar (RBC)		
Ready-to-use RBC medium	31.6 g/l	Carl Roth, Germany

### Morphological analyses and thermal preferences

Culture characteristics were studied on MEA and PDA. Detailed macroscopic characteristics were analysed using 4-weeks-old colonies of ex-type strains incubated at 15 °C. Detailed microscopic characteristics were analysed using about 8-weeks-old cultures grown at 6 °C on MEA. Fungal structures from the edge or surface of the cultures were placed in 80% lactic acid, heated to boiling point, cooled and examined under a Nikon Eclipse 80i light microscope at a magnification of × 1000. The microscopic structures were measured and photographed using NIS‐Elements BR 3.0 imaging software. Thermal preferences were assessed by measurement of the diameter of colonies of ex-type strains grown at 6 °C, 15 °C and 25 °C after 2, 4 and 8 weeks (Table 2). Thermal preferences of previously described *Rachicladosporium* species are adopted from the literature (Table 3).

**Table 2:** Thermal preferences of analysed *Rachicladosporium* strains. *R.* = *Rachicladosporium*.

Species	Strain	Colony diameter (in cm)					
		MEA			PDA		
		6 °C	15 °C	24 °C	6 °C	15 °C	24 °C
		2 weeks					
*R. europaeum*	G231	0.6	1.5	1.0	0.6	1.1	0.7
*R. ignacyi*	G398	0.7	1.5	0.5	0.7	1.2	0.4
*R. kajetanii*	G42	0.6	1.5	1.0	0.5	1.5	1.1
*R. silesianum*	G395	0.6	1.3	0.5	0.6	1.4	0.7
		4 weeks					
*R. europaeum*	G231	1.2	2.3	1.7	0.8	1.9	1.2
*R. ignacyi*	G398	1.5	2.4	0.8	1.6	2.3	0.6
*R. kajetanii*	G42	1.0	2.5	1.8	1.1	2.5	1.9
*R. silesianum*	G395	1.1	2.4	0.7	0.9	2.5	0.8
		8 weeks					
*R. europaeum*	G231	1.7	3.7	3.0	1.3	3.9	2.5
*R. ignacyi*	G398	2.3	3.8	1.6	2.8	4.3	1.7
*R. kajetanii*	G42	1.8	4.5	2.9	2.0	4.4	3.0
*R. silesianum*	G395	1.8	4.1	1.4	1.7	4.6	1.3

**Table 3:** Thermal preferences of previously reported *Rachicladosporium* species. *R.* = *Rachicladosporium*.

Species	Strain	Colony diameter (in cm; time of incubation; medium used)			References
		6˚C	15˚C	24˚C/25˚C	
*R. africanum*	CMW 39100	N/A	N/A	1.7 (10 d; MEA)	^10^
*R. alpinum*	CBS 136040	1.4 (2 mo; PDA)	3.9 (2 mo; PDA)	2.3 (2 mo; PDA)	^14^
*R. americanum*	CBS 124774	N/A	N/A	2 (10 d; MEA)	^8^
*R. antarcticum*	CCFEE 5527	1.3 (2 mo; PDA)	3.1 (2 mo; PDA)	no growth (2 mo; PDA)	^14^
*R. aridum*	MUT 6494 (= CCFEE 6514)	N/A	1.5 (12 wk; MEA)	N/A	^16^
*R. cboliae*	CBS 125424	N/A	N/A	1.5–3 (2 wk; OA, MEA, PDA)	^2^
*R. corymbiae*	CBS 145087	N/A	N/A	2.5 (2 wk; OA, MEA, PDA)	^13^
*R. eucalypti*	CPC 23241	N/A	N/A	1.2 (2 wk; OA, MEA, PDA)	^11^
*R. inconspicuum*	CBS 136043	1.7 (2 mo; PDA)	4.4 (2 mo; PDA)	1.6 (2 mo; PDA)	^14^
*R. iridis*	CBS 282.49	N/A	N/A	N/A	^12^
*R. luculiae*	CBS 121620	N/A	N/A	4 (1 mo; PDA)	^7^
*R. mcmurdoi*	CBS 119432	no growth (2 mo; PDA)	2.2 (2 mo; PDA)	no growth (2 mo; PDA)	^14^
*R. monterosanum*	CBS 137178	1 (2 mo; PDA)	1.6 (2 mo; PDA)	no growth (2 mo; PDA)	^14^
*R. paucitum*	CBS 136041	1.7 (2 mo; PDA)	4.5 (2 mo; PDA)	1.5 (2 mo; PDA)	^14^
*R. pini*	CBS 129525	N/A	N/A	1.5 (2 wk; OA, MEA, PDA)	^9^

CBS: Westerdijk Fungal Biodiversity Institute, Utrecht, Netherlands; CCFEE: Culture collection of fungi from extreme environments, Tuscia University, Viterbo, Italy; CMW: Culture collection of the Forestry and Agricultural Biotechnology Institute (FABI), University of Pretoria, Pretoria, South Africa; CPC: Culture collection of Pedro Crous, housed at the Westerdijk Fungal Biodiversity Institute, Utrecht, Netherlands; MUT: Mycotheca Universitatis Taurinensis, Department of Plant Biology of the University of Turin, Turin, Italy.

### DNA isolation, amplification and sequencing

Genomic DNA was extracted from pure colonies grown on MEA medium. Isolation was performed with DNeasy® Plant Mini Kit (Qiagen, Germany) according to manufacturer’s instructions. The amplification of nuc rDNA ITS1-5.8S-ITS2 (ITS) and nuc rDNA 28S D1–D2 (LSU) was performed using ITS1-ITS4 and LSU1Fd-LR5 primer pairs, respectively, or ITS1–LR5 primer set for fragment spanning ITS–LSU^2,34,35^. The RNA polymerase II (*rpb2*) gene was amplified using fRPB2-5F and RPB2-414R primers^36,37^. The amplification was performed in the 25 µl reaction mixture containing 2.5 μl of 10 × PCR Buffer and 25 mM MgCl_2_, 0.5 μl of 10 mM dNTPs and each 10 mM primers, 0.25 μl of 5 U/μl Taq DNA Polymerase (Sigma-Aldrich, St. Louis, Missouri, USA), 1 µl of genomic DNA. Cell culture grade water was used to fill up to a final volume. The amplification of ITS and LSU was performed as described by Czachura et al.^38^. The amplification of fragment spanning ITS–LSU and *rpb2* was performed with 3 min initial denaturation at 94 °C, followed by 35 cycles of denaturation at 94 °C for 45 s; the annealing of primers for 45 s at 50 °C (ITS–LSU) and 49 °C (*rpb2*); the elongation at 72 °C for 2 min (ITS–LSU) and 1.5 min (*rpb2*); the final extension at 72 °C for 10 min (ITS–LSU) and 6 min (*rpb2*). The PCR products were verified on 1% agarose gels and enzymatically cleaned using Exo-BAP Mix (EURx, Gdańsk, Poland). Bidirectional sequencing was carried out by Macrogen Europe B.V. (Amsterdam, the Netherlands). The sequencing reaction was performed using ITS1–ITS4, LSU1Fd–LR5 and fRPB2-5F and RPB2-414R primer pairs for, respectively, ITS, LSU and *rpb2* fragments^2,34,35^. All newly generated sequences were trimmed and assembled in Geneious Prime® 2020.0.4. Obtained sequences were deposited in NCBI’s GenBank nucleotide database (https://www.ncbi.nlm.nih.gov/genbank/).

### Phylogenetic analyses

Megablast searches in GenBank^39^ were used to find closest hits for the obtained sequences. Subsequently, newly generated sequences and reference sequences of representatives of the *Cladosporiales*, including all described *Rachicladosporium* species, were used to assemble a concatenated ITS, LSU, *rpb2* alignment and conduct phylogenetic analyses. *Pseudocercospora eucalyptorum* was used as an outgroup (Table 4).

**Table 4:** List of species, with country of origin, host/substrate, strain and GenBank accession numbers, used in phylogenetic analyses. Superscript T denotes ex type strain (epitype, holotype or neotype).

Species	Country	Host/substrate	Strain	GenBank acc. no		
				ITS	LSU	*rpb2*
*Cladosporium cladosporioides*	Germany	indoor air	CBS 112388^T^	NR_119839	KX286982	–
*Cladosporium colombiae*	Colombia	*Cortaderia* sp.	CBS 274.80B^T^	NR_119729	NG_069779	–
*Cladosporium ramotenellum*	United Kingdom	leaves of *Arundo*	CBS 170.54 (= AFTOL-ID 1289)	AY213640	DQ678057	–
*Cladosporium verrucocladosporioides*	South Korea	*Rhus chinensis*	CBS 126363^T^	MH863939	–	–
*Cryoendolithus antarcticus*	Antarctica	rock	CCFEE 5527^T^	NR_144970	KF309990	KF310043
*Cryoendolithus aridus*	Antarctica	rock	MUT 6494^T^ (= EXF163)	MW834577	–	–
*Cryoendolithus mcmurdoi*	Antarctica	rock	CBS 119432^T^ (= CCFEE 5211)	NR_144967	NG_059442	–
*Cryoendolithus monterosanus*	Italy	rock	CBS 137178^T^ (= CCFEE 5398)	NR_144968	NG_069061	KF310039
*Graphiopsis chlorocephala*	Germany	stems of *Paeonia officinalis*	CBS 121523^T^	MH863114	MH874669	–
*Pseudocercospora eucalyptorum*	South Africa	leaves of *Eucalyptus nitens*	CBS 110777^T^	NR_111057	KF901944	KF902305
*Rachicladosporium africanum*	South Africa	African baobab tree (*Adansonia*)	CMW 39100^T^	KP662111	–	–
*Rachicladosporium alpinum*	Italy	rock	CBS 136040^T^ (= CCFEE 5395)	NR_144965	KF310035	–
*Rachicladosporium americanum*	USA	leaf litter of unknown host	CBS 124774^T^	MH863412	NG_078630	MN829336
*Rachicladosporium cboliae*	USA	twig debris	CBS 125424^T^ (= CPC 14034)	NR_156538	NG_057851	LT799763
*Rachicladosporium corymbiae*	Ghana	leaf spots of *Corymbia citriodora*	CBS 145087^T^	NR_161143	NG_067850	–
*Rachicladosporium eucalypti*	Ethiopia	leaves of *Eucalyptus globulus*	CBS 138900^T^ (= CPC 23241)	NR_155718	NG_070537	–
*Rachicladosporium europaeum*	Poland	sooty mould community on *Symphoricarpos albus*	G231^T^	OR094691	OR094683	OR096695
*Rachicladosporium ignacyi*	Poland	sooty mould community on *Tilia cordata*	G24	OR094689	OR094681	OR096693
*Rachicladosporium ignacyi*	Poland	sooty mould community on *Betula pendula*	F95	OR094687	OR094679	OR096691
*Rachicladosporium ignacyi*	Poland	sooty mould community on *Pinus nigra*	G21	OR094688	OR094680	OR096692
*Rachicladosporium ignacyi*	Poland	sooty mould community on *Pinus nigra*	G398^T^	OR094694	OR094686	OR096698
*Rachicladosporium inconspicuum*	Italy	rock	CBS 136043^T^ (= CCFEE 5456)	NR_144966	NG_059443	KF310041
*Rachicladosporium iridis*	Netherlands	leaf of *Iris* *pseudacorus*	CBS 282.49^T^	NR_169891	EU167586	–
*Rachicladosporium kajetanii*	Poland	sooty mould community on *Rosa* sp.	G42^T^	OR094690	OR094682	OR096694
*Rachicladosporium luculiae*	New Zealand	leaf spots on *Luculia* sp.	CBS 121620^T^	NR_160222	MH874675	–
*Rachicladosporium paucitum*	Italy	rock	CBS 136041^T^ (= CCFEE 5458)	NR_144969	KF309988	–
*Rachicladosporium pini*	Netherlands	needles of *Pinus monophylla*	CBS 129525^T^	JF951145	MH876826	LT799764
*Rachicladosporium silesianum*	Poland	sooty mould community on *Pinus nigra*	G395^T^	OR094693	OR094685	OR096697
*Rachicladosporium silesianum*	Poland	sooty mould community on *Symphoricarpos albus*	G232	OR094692	OR094684	OR096696
*Toxicocladosporium banksiae*	Australia	leaves of *Banksia aemula*	CBS 128215^T^	NR_152322	NG_069077	LT799767
*Toxicocladosporium irritans*	Suriname	mouldy paint	CBS 185.58^T^	NR_152316	MH869283	–
*Toxicocladosporium strelitziae*	South Africa	leaves of *Strelitzia reginae*	CBS 132535^T^	NR_111765	NG_042687	–
*Trimmatostroma salinum*	Slovenia	saltern	CBS 100461^T^	NR_160204	NG_064176	–
*Verrucocladosporium carpobroti*	South Africa	leaves of *Carpobrotus quadrifidus*	CBS 146784^T^	NR_171765	NG_074493	–
*Verrucocladosporium dirinae*	United Kingdom	*Dirina massiliensis*	CBS 112794^T^	NR_152317	MH862899	–
*Verrucocladosporium visseri*	South Africa	*Carpobrotus edulis*	CPC 36317^T^ (= CBS 146046)	NR_166320	NG_068322	–

CBS: Westerdijk Fungal Biodiversity Institute, Utrecht, Netherlands; CCFEE: Culture collection of fungi from extreme environments, Tuscia University, Viterbo, Italy; CMW: Culture collection of the Forestry and Agricultural Biotechnology Institute (FABI), University of Pretoria, Pretoria, South Africa; CPC: Culture collection of Pedro Crous, housed at the Westerdijk Fungal Biodiversity Institute, Utrecht, Netherlands; MUT: Mycotheca Universitatis Taurinensis, Department of Plant Biology of the University of Turin, Turin, Italy.

–Indicates unavailable sequence.

The ITS, LSU and *rpb2* datasets were independently aligned using the MAFFT algorithm^40^ in Geneious Prime R11. The final phylogenetic tree was constructed using the concatenated alignment of these three genes. Phylogenetic analyses were carried out using RAxML-NG v. 1.1.1 and MrBayes v. 3.2.6^41,42^, respectively. For maximum likelihood (ML) and Bayesian inference (BI) analyses the best substitution models were selected using ModelTest-NG v. 0.2.0^43^. Maximum likelihood analysis was conducted with bootstrapping 1000 replicates. Bayesian inference was performed with the following parameter: 1 000 000 generations with trees sampled every 100th generation. The first 25% of trees were discarded as burn-in. Maximum likelihood bootstrap (MLB) support values above 70% and Bayesian posterior probabilities (BPP) above 0.95 were considered strongly supported. FigTree v1.4.3 was used for visualising the final phylogenetic tree.

## Results

In total, eight strains assigned to the genus *Rachicladosporium* were obtained from sooty mould communities in southern Poland. Amongst the analysed trees and shrubs, the *Rachicladosporium* strains were found only on leaves or needles of five species: *Betula pendula* (one strain), *Pinus nigra* (three strains), *Rosa* sp. (one strain), *Symphoricarpos albus* (two strains) and *Tilia cordata* (one strain). Morphological characteristics of isolated fungi are given in species descriptions. All tested strains grew in all analysed temperatures (6 °C, 15 °C and 25 °C) on both culture media (MEA and PDA), with the best growth rate observed at 15 °C (Table 2). The literature data of thermal preferences for previously described *Rachicladosporium* species, whenever available, showed that most of them grew at 25 °C, except *R. antarcticum*, *R. mcmurdoi* and *R. monterosanum* for which the growth at this temperature was not observed (Table 3).

### Phylogenetic analyses

Megablast searches in GenBank^39^ revealed similarities of the sequences of the studied strains to sequences of the *Rachicladosporium* species. The concatenated ITS, LSU, *rpb2* alignment contained sequences belonging to 32 species, including 31 species of the *Cladosporiales* and a member of the *Mycosphaerellales* (*Pseudocercospora eucalyptorum*) used as an outgroup. The alignment contained a length of 1744 characters (with gaps), including 678 bp for the rDNA ITS, 759 bp for the rDNA LSU and 307 bp for the *rpb2*. The best matching substitution models selected for single locus alignments in the ML analysis were as follows: TIM1ef + I + G4 for both ITS and LSU, and TrNef + G4, JC and TPM2uf + G4 for *rpb2* (three codons). The BI analysis was performed with the following substitution models: SYM + I + G4 for both ITS and LSU, and K80 + G4, JC and HKY + G4 for *rpb2* codons.

**Figure 1:**
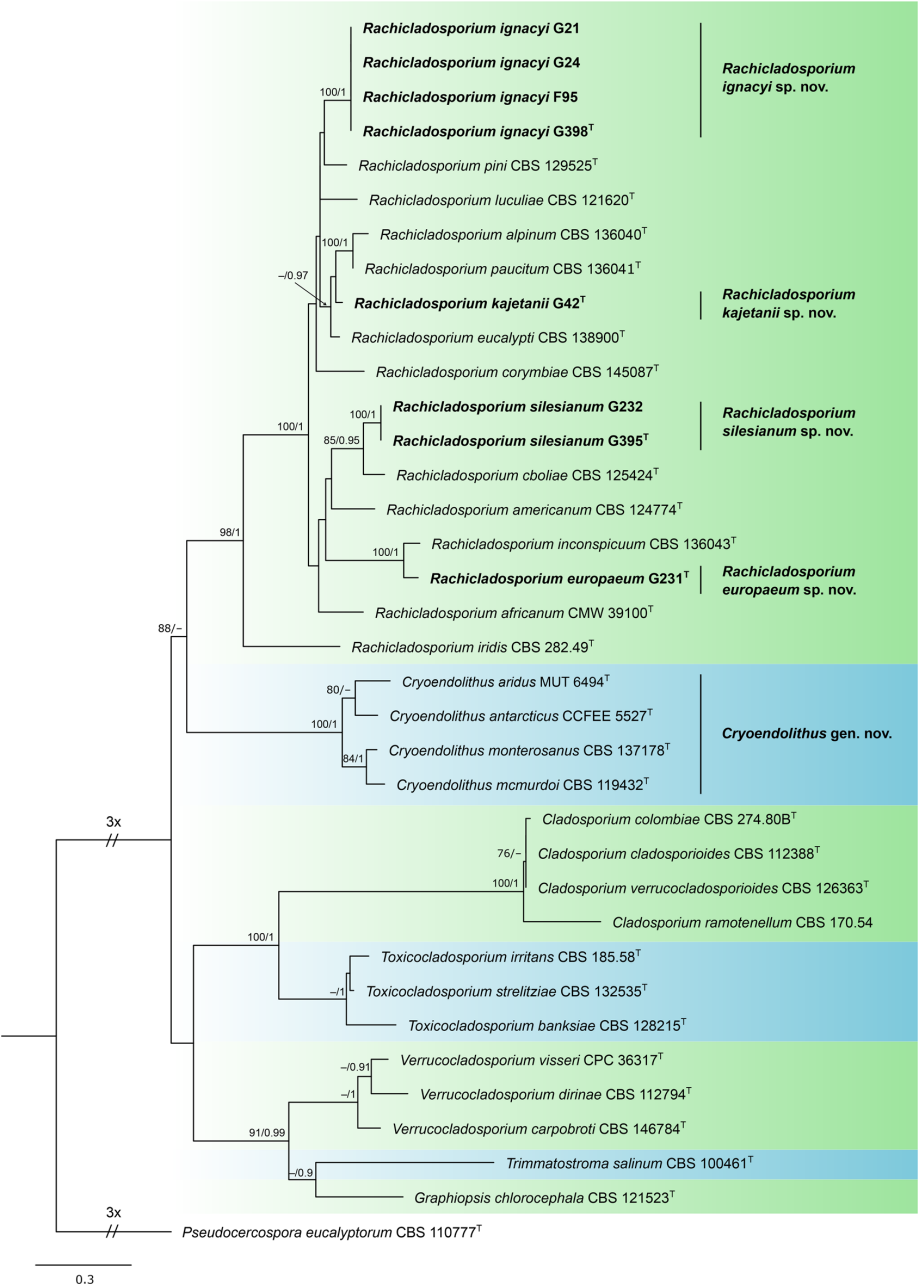
Phylogenetic tree of selected members of the *Cladosporiales*, including all sequenced *Rachicladosporium* species, obtained from a maximum likelihood analysis of the combined multi-locus alignment (ITS, LSU, *rpb2*). The positions of new *Rachicladosporium* species and new genus *Cryoendolithus* are indicated in bold. Ex-type cultures are indicated with superscript T. Numbers above branches indicate maximum likelihood bootstrap (MLB) support values > 70% and Bayesian posterior probabilities (BPP) > 0.9, respectively (MLB/BPP). *Pseudocercospora eucalyptorum* was used as an outgroup. The scale bar represents the expected number of changes per site.

ML and BI analyses resulted in similar tree topologies. The best scoring maximum likelihood phylogenetic tree is shown on Fig. 1. *Rachicladosporium* species were split into three groups: (i) main lineage containing type species, *Rachicladosporium luculiae*, (ii) single species lineage containing *Rachicladosporium iridis*, (iii) psychrophilic lineage containing three Antarctic and one Italian Alpine species. The main *Rachicladosporium* lineage was fully supported in both ML and BI analyses. *Rachicladosporium iridis* was strongly (MLB = 98%) or fully supported (BPP = 1) as a sister species to the main *Rachicladosporium* lineage. A clade grouping psychrophilic species (MBL = 100%, BPP = 1), formed a strongly supported (MLB = 88%) or not supported (BI analysis) sister lineage to the remaining two lineages.

The sooty mould strains formed four independent subclades in the main *Rachicladosporium* lineage. Sequences from cultures F95, G21, G24, G398 (described below as *R. ignacyi*) were almost identical in ITS (F95 differed in one bp from remaining strains) and identical in LSU and *rpb2* and formed a fully supported clade (MLB = 100%, BPP = 1) that was sister (though not supported) to *Rachicladosporium pini*. Sequences from cultures G232 and G395 (described below as *R. silesianum*) were identical in all analysed loci and formed a fully supported clade (MLB = 100%, BPP = 1) that was sister (and strongly supported, MLB = 85%, BPP = 0.95) to *Rachicladosporium cboliae*. Sequences from culture G42 (described below as *R. kajetanii*) were sister (though not supported) to *Rachicladosporium alpinum* and *R. paucitum*. Sequences from culture G231 (described below as *R. europaeum*) were sister (and fully supported, MLB = 100%, BPP = 1) to *Rachicladosporium inconspicuum*. Sequences of *R. europaeum* and *R. inconspicuum* differed in two base pairs (bp) in ITS, six bp in LSU and 39 bp in *rpb2*.

## Taxonomy

***Cryoendolithus*** Piątek, Stryjak-Bogacka & Czachura, gen. nov.

- MycoBank no. MB 850747
- Etymology: Named after its occurrence in icy cold and endolithic habitats.
- Description: Mycelium consisting of hyphae or hyphae and arthroconidia. Hyphae subhyaline, pale brown or dark brown, branched, usually torulose, rarely cylindrical. Arthroconidia dark brown, ellipsoidal, 0–1-septate. [adapted from 14].
- Type species: *Cryoendolithus mcmurdoi* (Selbmann & Onofri) Piątek, Stryjak-Bogacka & Czachura.
- Notes: *Cryoendolithus* is a new genus in the order *Cladosporiales*, morphologically similar to different rock inhabiting fungi, including some *Rachicladosporium* species, but phylogenetically distinct. It accommodates four endolithic and psychrophilic species known from extreme environments of Antarctica and the Alps. *Cryoendolithus* differs from *Rachicladosporium* s. str. in that its members are not able to grow at 25 °C.

***Cryoendolithus antarcticus*** (Egidi & Onofri) Piątek, Stryjak-Bogacka & Czachura, comb. nov.

- MycoBank no. MB 850748
- Basionym: *Rachicladosporium antarcticum* Egidi & Onofri, Fungal Diversity 65: 162 (2014).

***Cryoendolithus aridus*** (Selbmann & Coleine) Piątek, Stryjak-Bogacka & Czachura, comb. nov.

- MycoBank no. MB 850749
- Basionym: *Rachicladosporium aridum* Selbmann & Coleine, in Wijayawardene et al., Mycosphere 12(1): 1311 (2021).

***Cryoendolithus mcmurdoi*** (Selbmann & Onofri) Piątek, Stryjak-Bogacka & Czachura, comb. nov.

- MycoBank no. MB 850750
- Basionym: *Rachicladosporium mcmurdoi* Selbmann & Onofri, in Crous et al., Fungal Systematics and Evolution 3: 130 (2019).
- Synonym: *Rachicladosporium mcmurdoi* Selbmann & Onofri, Fungal Diversity 65: 159 (2014), nom. inval., art. 40.7 (Shenzhen).

***Cryoendolithus monterosanus*** (Isola & Zucconi) Piątek, Stryjak-Bogacka & Czachura, comb. nov.

- MycoBank no. MB 850751
- Basionym: *Rachicladosporium monterosanum* Isola & Zucconi, in Crous et al., Fungal Systematics and Evolution 3: 130 (2019).
- Synonym: *Rachicladosporium monterosium* Isola & Zucconi, Fungal Diversity 65: 161 (2014), nom. inval., art. 40.7 (Shenzhen).

**Figure 2:**
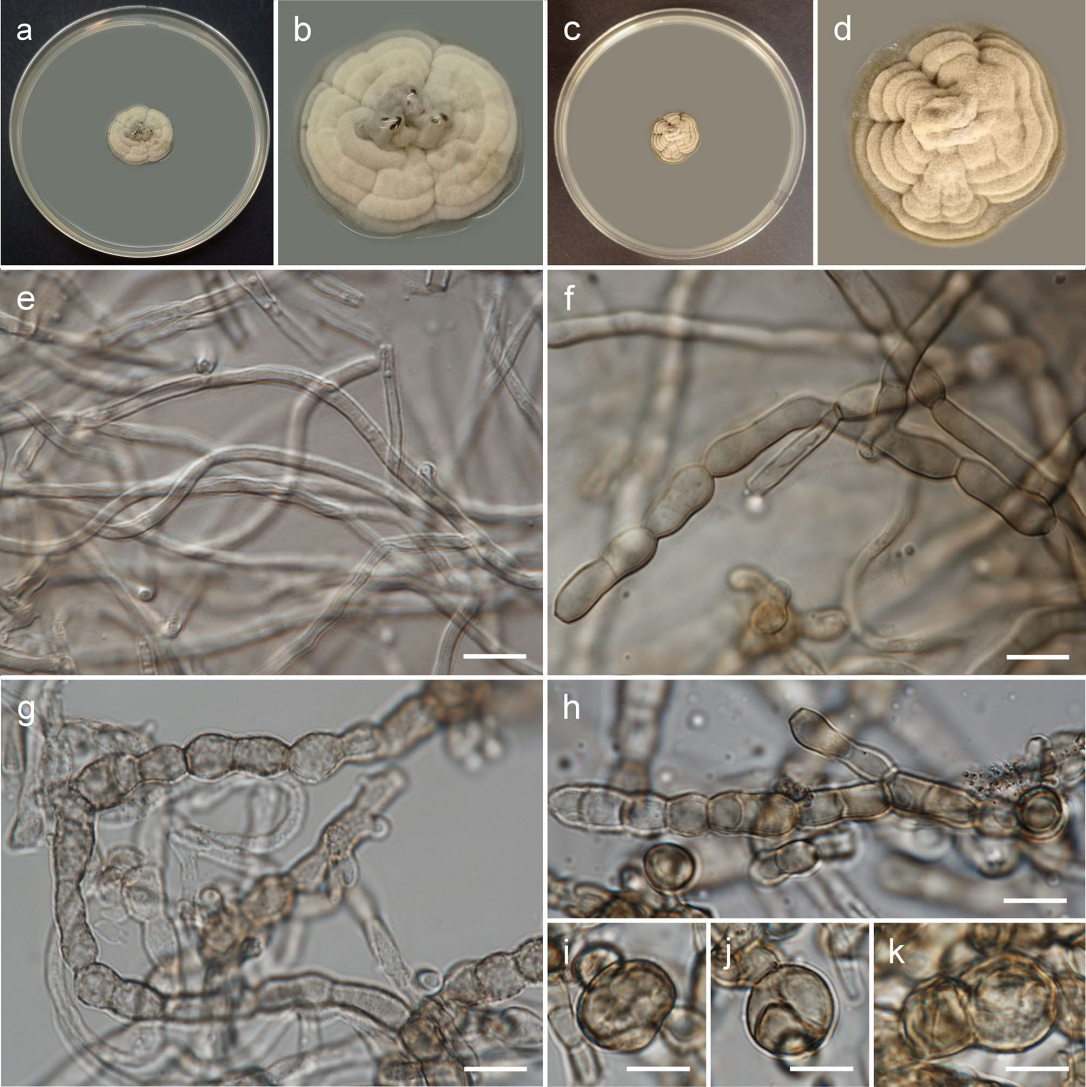
Morphology of *Rachicladosporium europaeum* Piątek, Stryjak-Bogacka & Czachura (strain G231): (**a**, **b**) general view and detailed view of upper side of colony on MEA after 4 weeks of growth at 15 °C; (**c**, **d**) general view and detailed view of upper side of colony on PDA after 4 weeks of growth at 15 °C; (**e**) hyphae; (**f**–**h**) terminal and intercalary arthroconidia; (**i**–**k**) muriformly septate chlamydospores. Scale bars: = 10 µm.

***Rachicladosporium europaeum*** Piątek, Stryjak-Bogacka & Czachura, sp. nov. Figure 2

- MycoBank no. MB 850752
- Etymology: Named after Europe, where the fungus was collected, by analogy to other described *Rachicladosporium* species (*R. africanum* from Africa, *R. americanum* from North America, and *R. antarcticum* from Antarctica).
- DNA barcodes (from type material): ITS (OR094691), LSU (OR094683), *rpb2* (OR096695).
- Description (on MEA): Mycelium composed of sparsely branched, septate, subhyaline or pale brown, smooth or finely verrucose, thin-walled, straight hyphae, 2.0–4.0 µm wide; hyphae develop into arthroconidia and chlamydospores. Arthroconidia, ellipsoid or broadly ellipsoid, rarely pyriform or elongated, subhyaline or pale brown, smooth, finely verrucose or verrucose, one-septate, rarely two-septate, 10.0–18.0 × 4.5–8.0 µm (up to 25 × 8.0 µm in young mycelium growing into medium), produced intercalary or terminally, in chains, sometimes branched. Chlamydospores sparse, globose, pale brown or brown, smooth, muriformly septate, 10.0–17.0 µm in diam.
- Culture characteristics: Colonies on MEA erumpent, spreading, with elevated and folded centre, olivaceous grey, forming concentric rings, reaching 23 mm diam after 4 weeks growth at 15 °C, surface with moderate aerial mycelium, margin smooth and lobate, concolours with the remaining part. Reverse dark olivaceous brown. Colonies on PDA erumpent, spreading, with elevated and folded centre, olivaceous grey, forming concentric rings, reaching 19 mm diam after 4 weeks growth at 15 °C, surface with moderate aerial mycelium, margin smooth and lobate, darker than the remaining part. Reverse dark olivaceous brown.
- Typus: **Poland**, Małopolska Province, Tarnów County: Tarnów–Gumniska-Zabłocie, municipal greenery, isolated from sooty mould community on *Symphoricarpos albus* leaves, 1 Oct. 2018, *leg. M. Piątek, W. Bartoszek & P. Czachura* (holotype: KRAM F-59829; ex type culture: G231 = CBS 150711).
- Notes: *Rachicladosporium europaeum* is phylogenetically closely related to *R. inconspicuum* but differs in having smooth to verrucose arthroconidia and muriformly septate chlamydospores. *R. inconspicuum*, isolated from rocks in the Italian Alps, produces only pale brown hyphae composed of cylindroid cells^14,15^.

**Figure 3:**
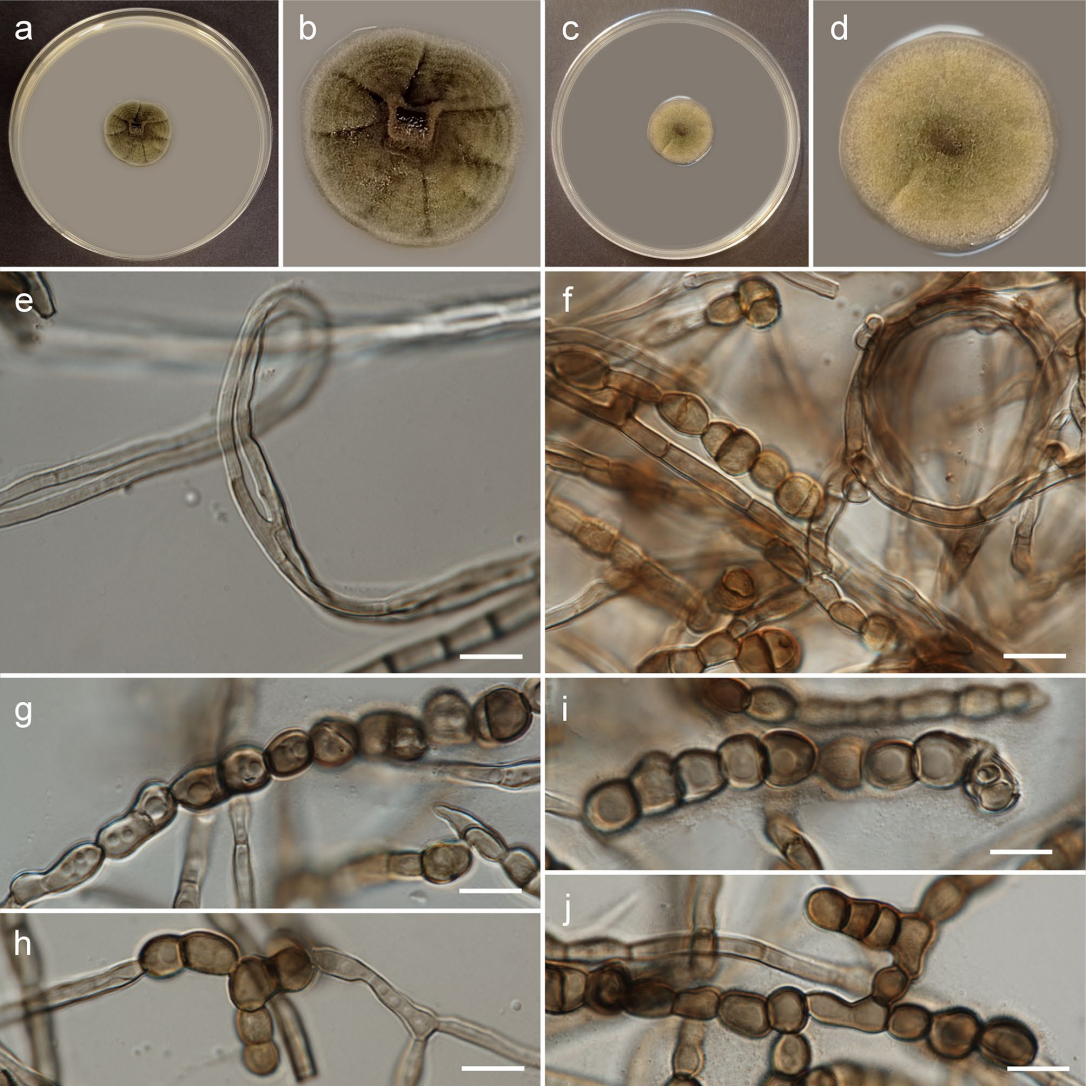
Morphology of *Rachicladosporium ignacyi* Piątek (strain G398): (**a**, **b**) general view and detailed view of upper side of colony on MEA after 4 weeks of growth at 15 °C; (**c**, **d**) general view and detailed view of upper side of colony on PDA after 4 weeks of growth at 15 °C; (**e**) anastomosing hyphae; (**f**) hyphae, some coiled, and arthroconidia; (**g**, **h**) arthroconidia; (**i**) chlamydospores; (**j**) chlamydospores and multiseptate conidium arising from reduced conidiophore. Scale bars: = 10 µm.

**Figure 4:**
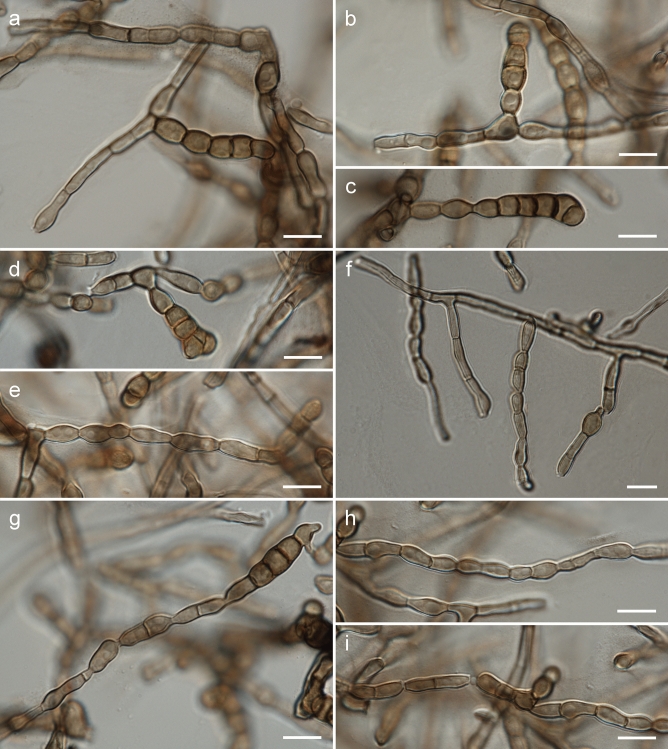
Morphology of *Rachicladosporium ignacyi* Piątek (strain G398): (**a**) multiseptate and one-septate conidia; (**b**–**d**) multiseptate conidia; (**e**–**f**) conidiophores and one-septate conidia; (**g**) one-septate conidia and one multiseptate conidium; (**h**, **i**) one-septate conidia. Scale bars: = 10 µm.

***Rachicladosporium ignacyi*** Piątek, sp. nov. Figs. 3, 4

- MycoBank no. MB 850753
- Etymology: Named after Ignacy Piątek, who often accompanied the first author in a collection of fungi, including one sample of this species.
- DNA barcodes (from type material): ITS (OR094694), LSU (OR094686), *rpb2* (OR096698). For the remaining barcodes see Table 4.
- Description (on MEA): Mycelium composed of sparsely branched, septate, subhyaline, pale brown or brown, smooth, thin-walled hyphae, 2.0–5.0 µm wide, sometimes coiled or anastomosing; hyphae develop into arthroconidia, chlamydospores and conidiophores with two types of conidia. Arthroconidia sparse, ellipsoid or broadly ellipsoid, rarely pyriform, pale brown or brown, smooth or rarely finely verrucose, one-septate, 8.0–17.0 × 5.5–8.5 µm, constricted at septa, thick-walled, produced intercalary or terminally, usually in chains. Chlamydospores sparse, globose, subglobose or broadly ellipsoid, pale brown or brown, smooth, nonseptate, 6.0–11.0 × 5.0–9.5 µm, thick-walled, produced intercalary or terminally, single or in chains. Conidiophores subcylindrical, pale brown, smooth, 0–2-septate, up to 20 µm long and 3.5 µm wide, or reduced to conidiogenous cells. Conidia multiseptate and one-septate. Multiseptate conidia cylindrical or fusiform, brown or dark brown, smooth or finely verrucose, 2–5-septate (occasionally with one longitudinal septa), 13.5–31.0 × 5.0–8.0 µm, constricted at septa, thick-walled, single. One-septate conidia subcylindrical, subhyaline, pale brown or brown, smooth or verrucose, one-septate (occasionally with two septa), 11.0–27.0 × 3.5–5.5 µm, sometimes constricted at septa, usually in chains.
- Culture characteristics: Colonies on MEA flat, spreading, with an elevated centre, greenish olivaceous, forming indistinct concentric rings, reaching 24 mm diam after 4 weeks growth at 15 °C, surface with abundant aerial mycelium, margin smooth and entire, lighter (whitish) than the remaining part. Reverse black. Colonies on PDA flat, spreading, pale greenish olivaceous, reaching 23 mm diam after 4 weeks growth at 15 °C, surface with abundant aerial mycelium, margin smooth and entire, lighter (whitish) than the remaining part. Reverse olivaceous brown.
- Typus: **Poland**, Małopolska Province, Tarnów County: Tarnów–Westerplatte, municipal greenery, isolated from sooty mould community on *Pinus nigra* needles, 1 Oct. 2018, *leg. M. Piątek, W. Bartoszek & P. Czachura* (holotype: KRAM F-59830; ex type culture: G398 = CBS 150712).
- Additional specimens examined: **Poland**, Podkarpackie Province, Rzeszów County: Rzeszów–Generała Władysława Andersa, municipal greenery (city park and surroundings), isolated from sooty mould community on *Tilia cordata* leaves, 17 Sept. 2018, *leg. M. Piątek, W. Bartoszek & P. Czachura* (KRAM F-59831; culture G24 = CBS 150713); **Poland**, Małopolska Province, Nowy Targ County: Jabłonka, garden, isolated from sooty mould community on *Betula pendula* leaves, 23 Jul. 2018, *leg. M. Piątek* (KRAM F-59832; culture F95 = CBS 150714); **Poland**, Małopolska Province, Tarnów County: Tarnów–Piaskówka, municipal greenery (city park and surroundings), isolated from sooty mould community on *Pinus nigra* needles, 1 Oct. 2018, *leg. M. Piątek, W. Bartoszek & P. Czachura* (KRAM F-59833; culture G21 = CBS 150715).
- Notes: *Rachicladosporium ignacyi* is phylogenetically closely related to *R. pini* but produces a complex set of fungal structures, including hyphae, arthroconidia, chlamydospores, and conidiophores with multiseptate and one-septate conidia. *Rachicladosporium pini* has been isolated from needles of *Pinus monophylla* in the Netherlands and forms a typical cladosporium-like morph with conidiophores and conidia in branched chains^9^.

**Figure 5:**
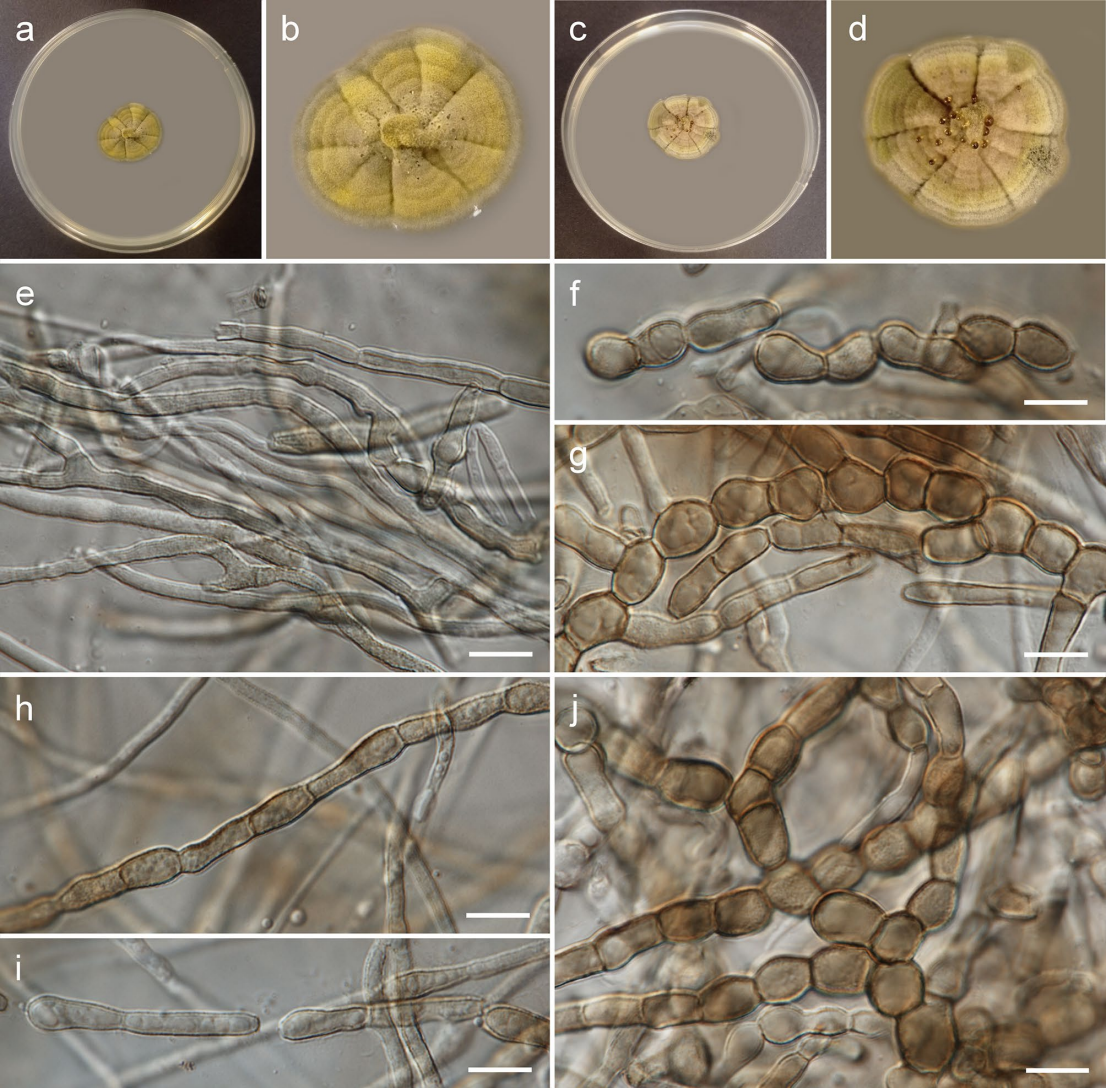
Morphology of *Rachicladosporium kajetanii* Piątek (strain G42): (**a**, **b**) general view and detailed view of upper side of colony on MEA after 4 weeks of growth at 15 °C; (**c**, **d**) general view and detailed view of upper side of colony on PDA after 4 weeks of growth at 15 °C; (**e**) hyphae; (**f**–**i**) arthroconidia; (**j**) chlamydospores. Scale bars: = 10 µm.

***Rachicladosporium kajetanii*** Piątek, sp. nov. Figure 5

- MycoBank no. MB 850754
- Etymology: Named after Kajetan Piątek, who often accompanied the first author in a collection of fungi, including a sample of this species.
- DNA barcodes (from type material): ITS (OR094690), LSU (OR094682), *rpb2* (OR096694).
- Description (on MEA): Mycelium composed of branched, septate, hyaline, subhyaline, pale brown or brown, smooth or verrucose, thin-walled, straight hyphae, 1.5–6.0 µm wide; hyphae develop into arthroconidia and chlamydospores. Arthroconidia, cylindrical, rarely ellipsoid, broadly ellipsoid or pyriform, subhyaline, pale yellow–brown or pale brown, finely verrucose or verrucose, rarely smooth, one-septate (occasionally with two septa), sometimes enclosed by mucilaginous sheath, 12.0–38.0 × 3.0–8.0 µm, often constricted at septa, produced by fragmenting hyphae, intercalary, rarely terminally or on side branches, usually in chains. Chlamydospores sparse, globose, subglobose or broadly ellipsoid, pale brown or brown, smooth or finely verrucose, non-septate, 7.0–11.0(− 11.0) × 6.0–9.0 µm in diam, produced intercalary, in chains.
- Culture characteristics: Colonies on MEA flat, spreading, with an elevated centre, greenish yellow, forming concentric rings, reaching 25 mm diam after 4 weeks growth at 15 °C, surface with moderate aerial mycelium, margin smooth and lobate, lighter (whitish) than the remaining part. Reverse olivaceous brown. Colonies on PDA flat, spreading, greenish olivaceous, forming concentric rings, reaching 25 mm diam after 4 weeks growth at 15 °C, surface with moderate aerial mycelium, margin smooth and lobate, lighter (whitish) than the remaining part. Reverse olivaceous brown.
- Typus: **Poland**, Małopolska Province, Nowy Targ County: Zakopane, garden, isolated from sooty mould community on *Rosa* sp. leaves, 7 Oct. 2018, *leg. M. Piątek* (holotype: KRAM F-59834; ex type culture: G42 = CBS 150716).
- Notes: *Rachicladosporium kajetanii* is phylogenetically closely related to *R. alpinum, R. eucalypti and R. paucitum* but is morphologically distinct. *Rachicladosporium alpinum* and *R. paucitum* have been isolated from rocks in the Italian Alps and form only subhyaline to pale brown hyphae^14,15^. *Rachicladosporium eucalypti* was described from the leaves of *Eucalyptus globulus* in Ethiopia and has a mycosphaerella-like sexual morph^11^.

**Figure 6:**
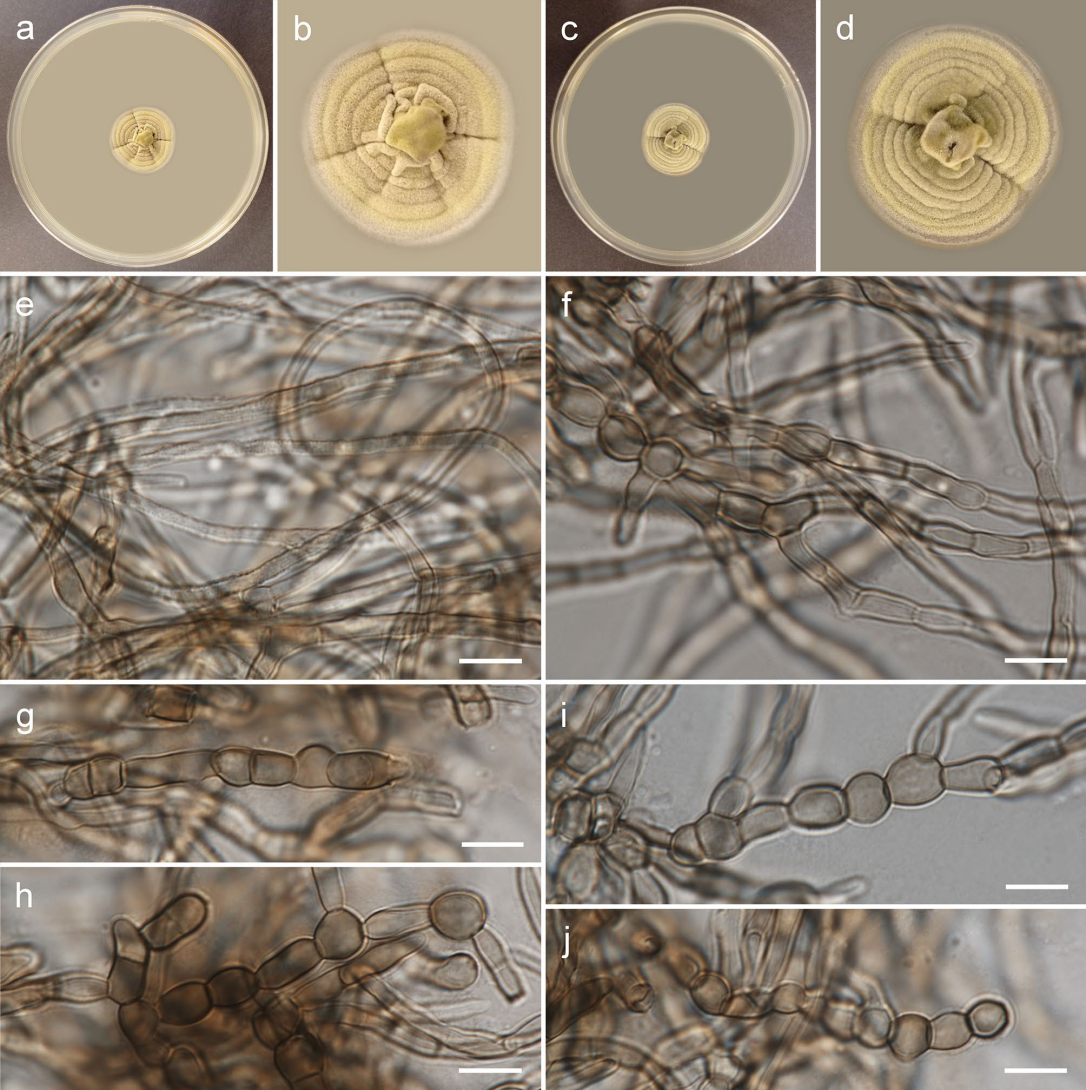
Morphology of *Rachicladosporium silesianum* Piątek, Stryjak-Bogacka & Czachura (strain G395): (**a**, **b**) general view and detailed view of upper side of colony on MEA after 4 weeks of growth at 15 °C; (**c**, **d**) general view and detailed view of upper side of colony on PDA after 4 weeks of growth at 15 °C; (**e**) hyphae; (**f**) hyphae and chlamydospores; (**g**) arthroconidia; (**h**) arthroconidia, chlamydospores and hyphae; (**i**, **j**) chlamydospores. Scale bars: = 10 µm.

***Rachicladosporium silesianum*** Piątek, Stryjak-Bogacka & Czachura, sp. nov. Figure 6

- MycoBank no. MB 850755
- Etymology: Name refers to the Silesia region in Poland, where the fungus was collected.
- DNA barcodes (from type material): ITS (OR094693), LSU (OR094685), *rpb2* (OR096697). For the remaining barcodes see Table 4.
- Description (on MEA): Mycelium composed of branched, septate, subhyaline, pale brown or brown, smooth or rarely finely verrucose, thin-walled, straight hyphae, 2.0–3.5 µm wide, usually constricted at septa, sometimes with swellings, up to 5 µm wide; hyphae develop into arthroconidia and chlamydospores. Arthroconidia sparse, ellipsoid or broadly ellipsoid, rarely pyriform, pale brown or brown, smooth, one-septate, 9.0–14.5 × 5.0–7.5 µm, produced intercalary or terminally, usually single. Chlamydospores abundant, globose, subglobose or broadly ellipsoid, pale brown or brown, smooth, nonseptate, 5.0–9.0 × 4.5–8.0 µm, produced intercalary or terminally, usually in chains.
- Culture characteristics: Colonies on MEA flat, spreading, with an elevated and folded centre, yellow olivaceous, forming concentric rings, reaching 24 mm diam after 4 weeks growth at 15 °C, surface with sparse aerial mycelium, margin smooth and entire, lighter (whitish) than the remaining part. Reverse olivaceous brown. Colonies on PDA flat, spreading, with an elevated and folded centre, yellow olivaceous, forming concentric rings, reaching 25 mm diam after 4 weeks growth at 15 °C, surface with sparse aerial mycelium, margin smooth and entire, lighter (whitish) than the remaining part. Reverse olivaceous brown.
- Typus: **Poland**, Silesian Province, Katowice County: Katowice-Brynów-Osiedle Zagrzebnioka, municipal greenery, isolated from sooty mould community on *Pinus nigra* needles, 10 Sept. 2018, *leg. M. Piątek, W. Bartoszek & P. Czachura* (holotype: KRAM F-59835; ex type culture: G395 = CBS 150717).
- Additional specimen examined: **Poland**, Silesian Province, Katowice County: Katowice-Bogucice, municipal greenery (city park and surroundings), isolated from sooty mould community on *Symphoricarpos albus* leaves, 10 Sept. 2018, *leg. M. Piątek, W. Bartoszek & P. Czachura* (KRAM F-59836; culture G232 = CBS 150718).
- Notes: *Rachicladosporium silesianum* is phylogenetically closely related to *R. cboliae* but differs in having only hyphae, arthroconidia and chlamydospores. *Rachicladosporium cboliae* has been isolated from twig litter in the USA and produces chlamydospores and cladosporium-like conidiophores with conidia^2^. Chlamydospores in *Rachicladosporium silesianum* are larger than those in *R. cboliae* (up to 6 µm in diam)^2^.

## Discussion

This study reports on so far unknown *Rachicladosporium* species isolated from sooty mould communities on the surface of the leaves or needles of woody plants in Poland. The habitat occupied by sooty moulds is an extreme environment, rich in sugars and poor in water^32^, from which none *Rachicladosporium* species has been recorded so far. *Rachicladosporium africanum* was described from black mould on the bark of baobab (*Adansonia digitata*) trees in Africa^10^. Indeed, fungi involved in the black mould on baobab trees were commonly considered to be “sooty moulds” but are different in that they penetrate infected tissues and cause host reaction^10^.

Eight isolated *Rachicladosporium* strains, obtained amongst hundreds of fungal strains isolated from sooty moulds, represented four species that were previously undescribed. Phylogenetically, they reside in the main *Rachicladosporium* lineage containing type species, *Rachicladosporium luculiae*. The morphology is complex in *Rachicladosporium ignacyi*, but simplified in the remaining newly described species that contain only hyphae, chlamydospores and arthroconidia. Our hypothesis that they are distinct species was supported by molecular data. According to the phylogenetic analyses they form independent subclades scattered over the subclades of *Rachicladosporium* species with completely different morphologies (Fig. 1; see notes in the species descriptions). Although described species are probably not host-specific, most strains were obtained from *Pinus nigra* and the types of *Rachicladosporium ignacyi* and *R. silesianum* are from this host plant. Another species, *Rachicladosporium pini*, has been previously described from needles of *Pinus monophylla*^9^. Unclassified species were also reported as endophytes of *Pinus densiflora*, *P. koraiensis*, *P. rigida*, *P. thunbergii* and *P. wallichiana*^21,23^. This indicates that *Pinus* may be the favourite genus for *Rachicladosporium* and harbour further undescribed species.

The simple morphology in the three *Rachicladosporium* species described here from sooty mould communities is morphologically convergent with, for example, rock inhabiting fungi, which often produce similar morphological structures^14,44,45^. Sooty moulds and rock inhabiting fungi are exposed to low water availability, ultraviolet radiation, temperature fluctuations and limited amount of nutrients^46^, and probably the development of similar simple morphologies is an adaptation to occurrence in similar extreme environments. In fact, some *Rachicladosporium* species were described from rocks^14,16^. These include *R. alpinum*, *R. inconspicuum* and *R. paucitum* from the main *Rachicladosporium* lineage and *R. antarcticum*, *R. aridum*, *R. mcmurdoi* and *R. monterosanum* from the psychrophilic lineage. Interestingly, *R. europaeum* and *R. kajetanii* described here from sooty mould communities are closely related to *R. inconspicuum* and *R. alpinum*/*R. paucitum*, respectively. Close relationships between sooty mould and rock inhabiting fungi were already reported for the orders *Capnodiales* (s.l.) and *Chaetothyriales*^32,47^. It is considered that rock inhabiting fungi evolved from sooty moulds. Honeydew dropped from the plants into the rocks may have been the vehicle in their evolution^32^. This is, however, still a poorly known phenomenon deserving in-depth analyses.

The main *Rachicladosporium* lineage contains species that are able to grow at 25 °C. This refers also to rock inhabiting fungi, isolated from the Alps, nested within this lineage. By contrast, four cold adapted, rock inhabiting species from Antarctica (three species) and Italian Alps (one species) that form psychrophilic lineage, do not grow at this temperature (Table 3)^14,16^. The phylogenetic distance of this lineage is comparable between genetic distances in related genera of the *Cladosporiales*. Therefore, the reassessment of published morphological characters and thermal preferences^14,16^ together with molecular phylogenetic analyses conducted in this study give arguments to include them in a distinct genus, named here as *Cryoendolithus*. *Rachicladosporium iridis* may belong to yet another undescribed genus to be separated from the *Rachicladosporium* s. str. Since it forms a single species lineage, the eventual reclassification into separate genus needs more species and sequences.

In summary, the results of this work expand the knowledge on the diversity and ecological preferences of the genus *Rachicladosporium*. It also excludes from the genus *Rachicladosporium* cold adapted species and classifies them in the distinct genus *Cryoendolithus*. It is likely that further species will be described in this genus as this may be deduced from the reports of unclassified species available in the literature.

## Data Availability

The data that support the findings of this study are available in GenBank (https://www.ncbi.nlm.nih.gov/genbank/) and in culture collections and fungal herbarium, as shown in Table 4 and the text.
